# Diagnostic Accuracy of Medical Imaging–Based Artificial Intelligence for Osteonecrosis of the Femoral Head: Systematic Review and Meta-Analysis

**DOI:** 10.2196/95648

**Published:** 2026-08-20

**Authors:** FeiLong Lu, LiRong Wang, Wenbin Zhang, YuLin Ma, JingYuan Tian, YiMei Hu

**Affiliations:** 1 School of Clinical Medicine, Chengdu University of Traditional Chinese Medicine, Chengdu, Sichuan, China; 2 Department of Orthopedics, Qionglai Hospital of Traditional Chinese Medicine, Qionglai, Sichuan, China; 3 Department of Orthopedics, Affiliated Hospital of Chengdu University of Traditional Chinese Medicine, 37 Shi'erqiao Road, Chengdu, Sichuan Province, 610075, China, 86 18908041502

**Keywords:** osteonecrosis of the femoral head, artificial intelligence, deep learning, machine learning, diagnostic imaging, meta-analysis

## Abstract

**Background:**

Osteonecrosis of the femoral head (ONFH) is a common cause of hip disability in clinical practice. Early and accurate diagnosis can delay or even halt disease progression. In recent years, AI models based on medical imaging have been increasingly applied to the diagnosis of ONFH; however, a systematic evaluation of their diagnostic accuracy remains lacking.

**Objective:**

This study aims to synthesize the overall diagnostic accuracy of medical imaging-based AI models for ONFH and to inform clinical decision-making.

**Methods:**

This systematic review was conducted in accordance with the PRISMA-DTA (Preferred Reporting Items for Systematic Reviews and Meta-Analyses of Diagnostic Test Accuracy Studies) guidelines and was prospectively registered in PROSPERO (CRD420261307216). We searched PubMed, Embase, Cochrane Library, and Web of Science up to March 8, 2026. Studies developing or validating AI models for ONFH diagnosis using imaging data were eligible. Risk of bias was assessed using the QUADAS-2 tool. Sensitivity, specificity, positive likelihood ratio (PLR), negative likelihood ratio (NLR), and diagnostic odds ratio (DOR) were pooled using a bivariate mixed-effects model, and a summary receiver operating characteristic (SROC) curve was constructed. Subgroup analyses were stratified by imaging modality (x-ray vs MRI), disease stage (early-stage ONFH vs all-stage ONFH), diagnostic criteria (Association Research Circulation Osseous [ARCO] staging vs other criteria), control group type (healthy controls vs disease controls), validation method (internal validation vs external validation), center type (single-center vs multicenter), and model type (deep learning vs machine learning). Meta-regression was performed to quantify the contribution of each covariate to between-study heterogeneity. Sensitivity analysis and Deeks asymmetry test assessed the robustness of the results and publication bias. Clinical utility was evaluated using the Fagan nomogram.

**Results:**

A total of 12 studies comprising 16,189 hip joints were included. The pooled sensitivity was 0.91 (95% CI 0.87‐0.95), the pooled specificity was 0.95 (95% CI 0.93‐0.96), and the SROC AUC was 0.97 (95% CI 0.95‐0.98). Substantial between-study heterogeneity was observed (*I*²=72%, 95% CI 38%‐100%). Subgroup analysis showed that MRI-based models yielded a higher diagnostic odds ratio (DOR; 382, 95% CI 220‐665) than x-ray-based models (106, 95% CI 60‐190), while models that underwent external validation had a lower DOR (129, 95% CI 51‐329) than those with only internal validation (230, 95% CI 104‐510). Meta-regression identified imaging modality as the primary source of heterogeneity, explaining 92.1% of the between-study variance.

**Conclusions:**

AI models demonstrate high diagnostic accuracy in imaging-based ONFH diagnosis. However, the current evidence is constrained by the limited number of included studies, predominantly retrospective designs, and a lack of adequate external validation, and should therefore be interpreted with caution. Future research should adopt multicenter prospective designs, standardize reference standards, and implement rigorous external validation to facilitate clinical translation.

## Introduction

Osteonecrosis of the femoral head (ONFH) is a condition that leads to hip joint dysfunction. Its underlying pathology is the death of bone cells from ischemia following disruption of the blood supply. Approximately 80% of patients eventually develop femoral head collapse, with the majority requiring total hip arthroplasty (THA), which imposes a substantial burden on both patients and health care systems [1,2]. The precise etiology of ONFH remains unclear; established risk factors include trauma, corticosteroid use, chronic alcohol consumption, and genetic factors [3-6]. Epidemiological data indicate that ONFH is more prevalent in men over 40 years of age, with an estimated 20,000 to 30,000 new cases diagnosed annually in the United States. The disease burden is considerably greater in China, where 75,000 to 150,000 new cases occur each year, with a cumulative patient population of 8.12 million, and the incidence has been rising since the COVID-19 pandemic [7-9]. Early and accurate diagnosis is directly linked to the choice of hip-preserving treatment and plays a decisive role in patient recovery outcomes. Precollapse intervention is therefore particularly critical: timely management can effectively delay the need for surgery, and a substantial proportion of patients may thus avoid joint replacement [10-12].

Clinicians typically rely on x-ray for the initial screening of ONFH. This modality is cost-effective and available in most health care facilities. However, x-ray is unreliable for detecting early-stage lesions, with a reported sensitivity of only 50%‐70%. Prior to femoral head collapse, it is often difficult for physicians to identify abnormalities on x-ray images [13]. Magnetic resonance imaging (MRI) is currently the core diagnostic modality for this condition, as the characteristic band-like sign of the necrotic region is readily identifiable on MR images. Nevertheless, the widespread adoption of MRI is constrained by several practical factors. MRI examinations are relatively time-consuming. The high cost imposes a financial burden on patients, and contraindications such as implanted devices preclude some individuals from undergoing the scan. These limitations make MRI difficult to adopt as a routine screening tool [14,15]. Moreover, the level of clinician experience substantially influences manual image interpretation; different readers may arrive at divergent conclusions when reviewing the same imaging data, and this subjectivity leads to suboptimal interreader consistency. Clinical data indicate that the initial misdiagnosis rate of ONFH reaches 20% to 30%, with particularly high rates in the early disease stages, where the occult nature of early pathological changes markedly increases the difficulty of manual detection [16,17].

AI technology has been widely applied in medical image analysis, with models capable of automatically extracting deep features from images to provide objective references for diagnosis [18,19]. Khan et al [20] proposed an architecture termed Progressive Residual Multi-Class Support Vector Machine-Net (PRMS-Net), a model that combines progressive residual networks with ResNet-50, which can automatically identify subtle lesions in heatmaps to assist breast cancer screening. Lee et al [21] developed a deep learning-based image assessment tool that evaluates knee osteoarthritis severity by measuring joint space width. In the ONFH domain, multiple AI models have been proposed for tasks such as lesion detection and disease staging [22,23]. However, the imaging modalities, algorithmic architectures, and diagnostic criteria vary considerably across studies, as do the validation approaches. These inconsistencies limit the clinical translation of this technology, making it difficult to deploy AI systems in real-world medical settings.

Therefore, this study conducted a systematic review and subgroup analysis of the accuracy of medical imaging-based AI technologies in the diagnosis and staging of ONFH, aiming to provide orthopedic surgeons and radiologists with evidence for evaluating the reliability of AI-assisted diagnosis.

## Methods

### Study Registration

This systematic review was conducted in accordance with the PRISMA-DTA (Preferred Reporting Items for Systematic Reviews and Meta-Analyses of Diagnostic Test Accuracy) statement [24-26] and was prospectively registered in the International Prospective Register of Systematic Reviews (PROSPERO; CRD420261307216). The PRISMA-DTA main checklist and abstract checklist are provided in Checklist 1 and Checklist 2, respectively.

### Data Sources and Search Strategy

A systematic search was performed in four databases, PubMed, Embase, Cochrane Library, and Web of Science, from database inception to March 8, 2026. The search strategy combined MeSH terms with free-text keywords, including “osteonecrosis of the femoral head,” “avascular necrosis of the femoral head,” “artificial intelligence,” “deep learning,” “machine learning,” “convolutional neural network,” “radiomics,” “diagnosis,” and “diagnostic imaging.” The complete search strategy for each database is detailed in Multimedia Appendix 1. Gray literature and trial registries were not searched, as only peer-reviewed original studies were considered eligible. The reference lists of all included studies and relevant reviews were manually screened to identify any potentially eligible studies missed during the database search.

### Inclusion and Exclusion Criteria

The inclusion criteria were as follows: (1) patients with ONFH confirmed by a reference standard, without restrictions on age, sex, disease stage, or etiology; (2) studies developing or validating deep learning (DL) or machine learning (ML) models based on medical imaging, with ONFH as the diagnostic task; (3) a reference standard defined as MRI-based diagnosis, histopathological confirmation, comprehensive clinical diagnosis, or diagnosis based on staging systems such as the Association Research Circulation Osseous (ARCO) staging, Ficat, or modified Ficat-Arlet classification; (4) study designs including cohort, case-control, or cross-sectional studies; (5) studies providing, or permitting the reconstruction of, a 2×2 diagnostic contingency table from which true positive (TP), false positive (FP), false negative (FN), and true negative (TN) values could be derived using reported sensitivity, specificity, accuracy, positive predictive value (PPV), negative predictive value (NPV), *F_1_*-score, confusion matrix, or area under the receiver operating characteristic curve (AUC); (6) availability of the full text.

The exclusion criteria were as follows: (1) studies that performed image segmentation only without constructing a complete diagnostic model; (2) studies from which a 2×2 diagnostic contingency table could not be extracted or reconstructed, including those reporting AUC alone without sensitivity or specificity data, where the primary data could not be obtained through reasonable means; (3) nonoriginal research, including reviews, systematic reviews, meta-analyses, conference abstracts, case reports, expert opinions, editorials, and letters; (4) animal studies or in vitro studies.

### Literature Screening and Data Extraction

All retrieved records were imported into Zotero (version 7.0.32; Corporation for Digital Scholarship), and duplicates were removed, followed by manual verification. Two reviewers (LF and WL) independently performed the initial screening by reviewing titles and abstracts to exclude studies clearly failing to meet the inclusion criteria. Full texts of potentially eligible studies were retrieved and independently assessed by the same 2 reviewers (LF and WL) to determine final inclusion. The screening process was documented in accordance with the PRISMA-DTA flowchart, with the number of excluded studies and reasons for exclusion recorded at each stage. Any disagreement between the 2 reviewers (LF and WL) regarding the inclusion of a study was resolved through discussion with a third reviewer (ZW). Data extraction was performed using a prespecified standardized electronic form, which captured the following information: first author, year, country, region, primary affiliation, study design, center type, reference standard, staging system, disease stage, imaging modality, model type, specific algorithm, validation method, control group type, total sample, ONFH cases, control cases, age, sex, comparison with clinicians, and key finding (AI vs clinicians). For diagnostic accuracy metrics, TP, FP, FN, and TN counts were extracted or derived for each study. When 2×2 contingency table data were not directly reported, TP was estimated as sensitivity × number of ONFH cases, FN as number of ONFH cases−TP, TN as specificity × number of controls, and FP as number of controls−TN, with fractional values rounded to the nearest integer. If a study reported the diagnostic performance of multiple AI models, the model recommended by the authors or the best-performing model was included in the primary analysis to avoid unit-of-analysis error. If a study reported both internal and external validation results, external validation data were preferentially selected to provide a more conservative estimate of model generalizability. If a study included multiple validation datasets of the same validation type, the best-performing model was selected.

### Risk-of-Bias Assessment

The methodological quality of the included studies was assessed using the Quality Assessment of Diagnostic Accuracy Studies-2 (QUADAS-2) tool [27]. Two reviewers (LF and WL) independently evaluated the risk of bias and applicability across the following domains: patient selection, index test, reference standard, and flow and timing. In accordance with the QUADAS-2 framework, if any signaling question within a domain was rated as “high risk,” the domain was judged as high risk overall; each domain was ultimately classified as “high risk,” “low risk,” or “unclear.” Consistent with the QUADAS-2 guidelines, no summary score was calculated. Any disagreement between the 2 reviewers (LF and WL) was resolved through discussion with a third reviewer (ZW). The results of the assessment were visualized using the Risk-of-Bias Visualization tool (Robvis) [28].

### Statistical Analysis

All statistical analyses were performed in Stata 17.0 (StataCorp LLC), using the “midas” module to pool diagnostic accuracy data under a bivariate random-effects model. Two-by-two contingency table data were extracted directly or derived from reported sensitivity, specificity, and sample sizes when not explicitly provided. Zero cells were handled without continuity correction, as the bivariate random-effects model possesses favorable statistical properties for sparse data, with extreme values managed through the internal algorithms of the “midas” module during model fitting. Pooled estimates included sensitivity, specificity, positive likelihood ratio (PLR), negative likelihood ratio (NLR), and diagnostic odds ratio (DOR), with a summary receiver operating characteristic (SROC) curve constructed. By jointly modeling the within-study and between-study correlations of sensitivity and specificity, the bivariate model produces more accurate pooled estimates and associated uncertainty than univariate pooling approaches. Between-study heterogeneity was assessed using the Cochran Q test (significance level set at 0.10), with the *I*² statistic quantifying the degree of variability: *I*²>50% was considered indicative of moderate heterogeneity, and *I*²>75% indicative of substantial heterogeneity. Subgroup analyses were stratified by imaging modality (x-ray vs MRI), disease stage (early-stage ONFH vs all-stage ONFH), diagnostic criteria (ARCO staging vs other criteria), control group type (healthy controls vs disease controls), validation method (internal validation vs external validation), center type (single-center vs multicenter), and model type (DL vs ML). Differences in effect sizes across subgroups were explicitly compared. Multivariable meta-regression was performed using the logarithm of the diagnostic odds ratio as the dependent variable, with imaging modality and validation method entered as core covariates, and the Knapp–Hartung method applied to adjust for small-sample bias. The regression coefficient and *R*² reflected the proportion of heterogeneity explained by each covariate. Leave-one-out sensitivity analysis was conducted by iteratively excluding each study to verify the robustness of the pooled results, ensuring that no single study disproportionately influenced the overall conclusions. Publication bias was assessed using Deeks asymmetry test. A *P* value of <.05 was considered indicative of significant publication bias; for subgroups in which bias was detected, the trim-and-fill method was applied to correct for funnel plot asymmetry and evaluate its impact on the pooled effect estimates. Clinical utility was assessed using the Fagan nomogram, in which the overall disease prevalence in each analysis group served as the pretest probability, and the posttest probability was computed by integrating the pooled PLR and NLR, illustrating the influence of a positive or negative test result on the diagnostic probability and assessing the potential clinical utility of AI models in decision-making.

### Ethical Considerations

Not applicable. This study is a systematic review and meta-analysis and did not involve direct research on human or animal participants.

## Results

### Literature Screening Process and Results

**Figure 1.**
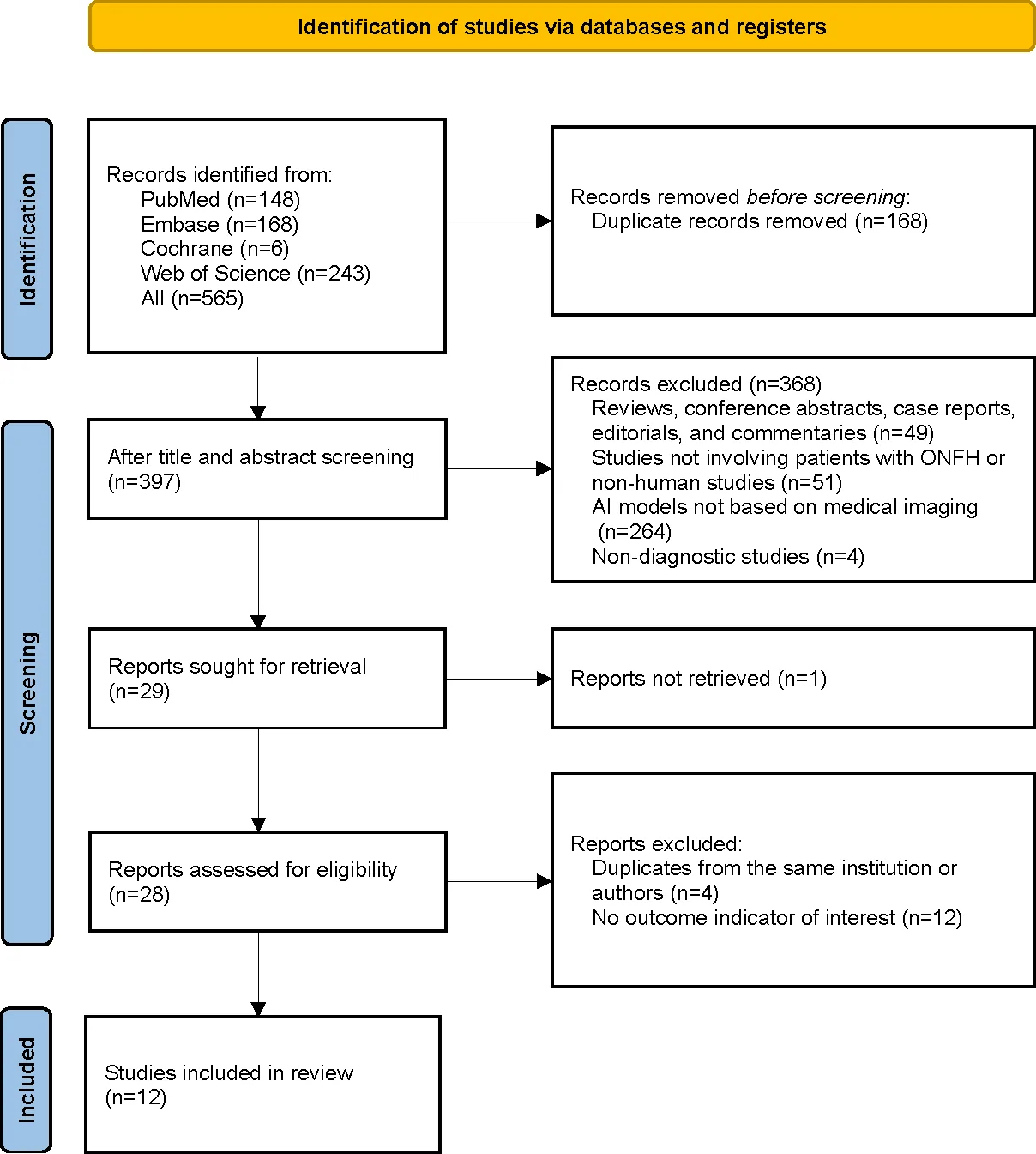
PRISMA (Preferred Reporting Items for Systematic Reviews and Meta-Analyses) flow diagram of the literature screening process.

A total of 565 records were initially retrieved from four databases: PubMed (n=148), Embase (n=168), Web of Science (n=243), and Cochrane Library (n=6). After deduplication, 397 records remained. Title and abstract screening excluded 368 records, including reviews, conference abstracts, case reports, and commentaries (n=49); studies not involving ONFH or nonhuman subjects (n=50); studies using AI models not based on medical imaging (n=264); and nondiagnostic studies (n=4). Full texts of the remaining 29 records were sought for further assessment. One record was inaccessible, leaving 28 studies for full-text review. Of these, 16 were excluded: 12 with incomplete diagnostic accuracy data and 4 due to duplicate reporting from the same institution or research team. Ultimately, 12 studies [29-40] were included in the qualitative synthesis. The study screening process is illustrated in Figure 1.

### Basic Characteristics and Risk of Bias of Included Studies

All 12 studies [29-40] were retrospective in design, including 11 cohort studies [29-36,38-40] and one case-control study [37], published between 2019 and 2026. Eight studies [29-32,34,35,37,38] originated from China, 2 studies [36,40] from South Korea, and one each from Iran [33] and Greece [39]. Regarding study design, 6 were single-center studies [29,31,36-39], and 6 were multicenter studies [30,32-35,40]. In terms of diagnostic criteria, 5 studies [30,34,35,37,40] used the ARCO staging system, while 7 studies [29,31-33,36,38,39] used other reference standards, including the Ficat staging system, the modified Ficat-Arlet staging system, comprehensive clinical diagnosis, and MRI-based diagnosis. With respect to disease stage, 6 studies [30,32,34-37] focused on early-stage ONFH, and 6 studies [29,31,33,38-40] included patients across all ONFH stages. Concerning imaging modality, 6 studies [29,30,33,36,38,40] were based on x-ray and 6 studies [31,32,34,35,37,39] on MRI. Model type was predominantly DL (n=9) [29,30,32,33,35-38,40], with ML accounting for 3 studies [31,34,39]. In terms of validation method, 7 studies [29,31,33,36-39] used internal validation, and 5 studies [30,32,34,35,40] used external validation. With respect to the control group, 8 studies [29-31,33,36,38-40] used healthy controls, and 4 studies [32,34,35,37] used disease controls, including osteoarthritis, transient osteoporosis, and tumors. Collectively, the 12 studies [29-40] comprised 16,189 hip joint images, of which 9253 were from patients with ONFH. In addition, 11 studies [30-40] compared the performance of AI models with that of clinicians (Multimedia Appendix 2).

**Figure 2.**
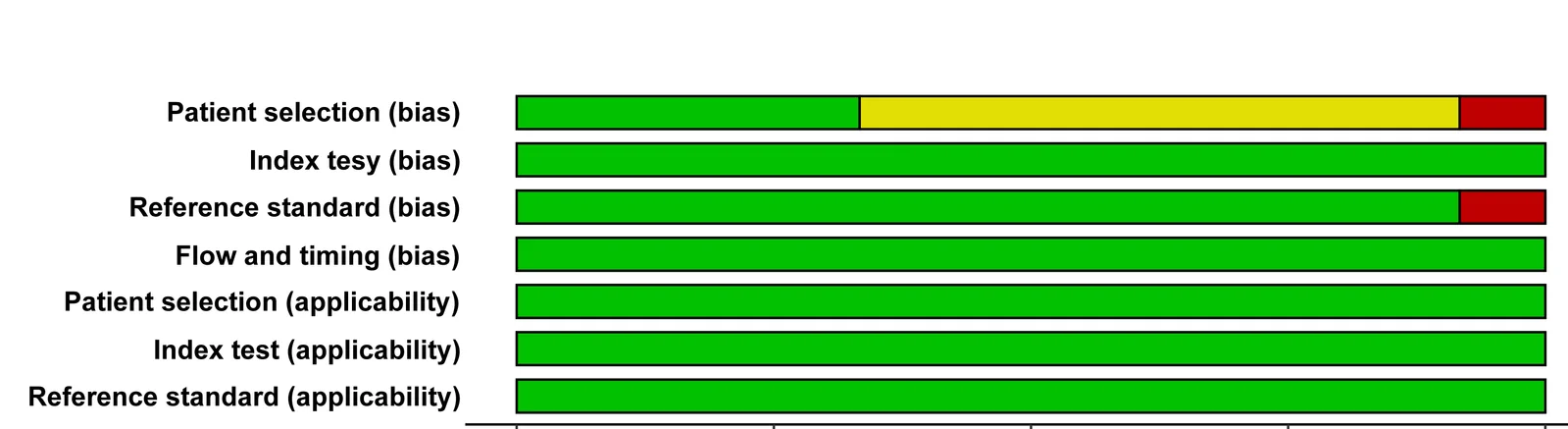
Summary plot of quality assessment.

**Figure 3.**
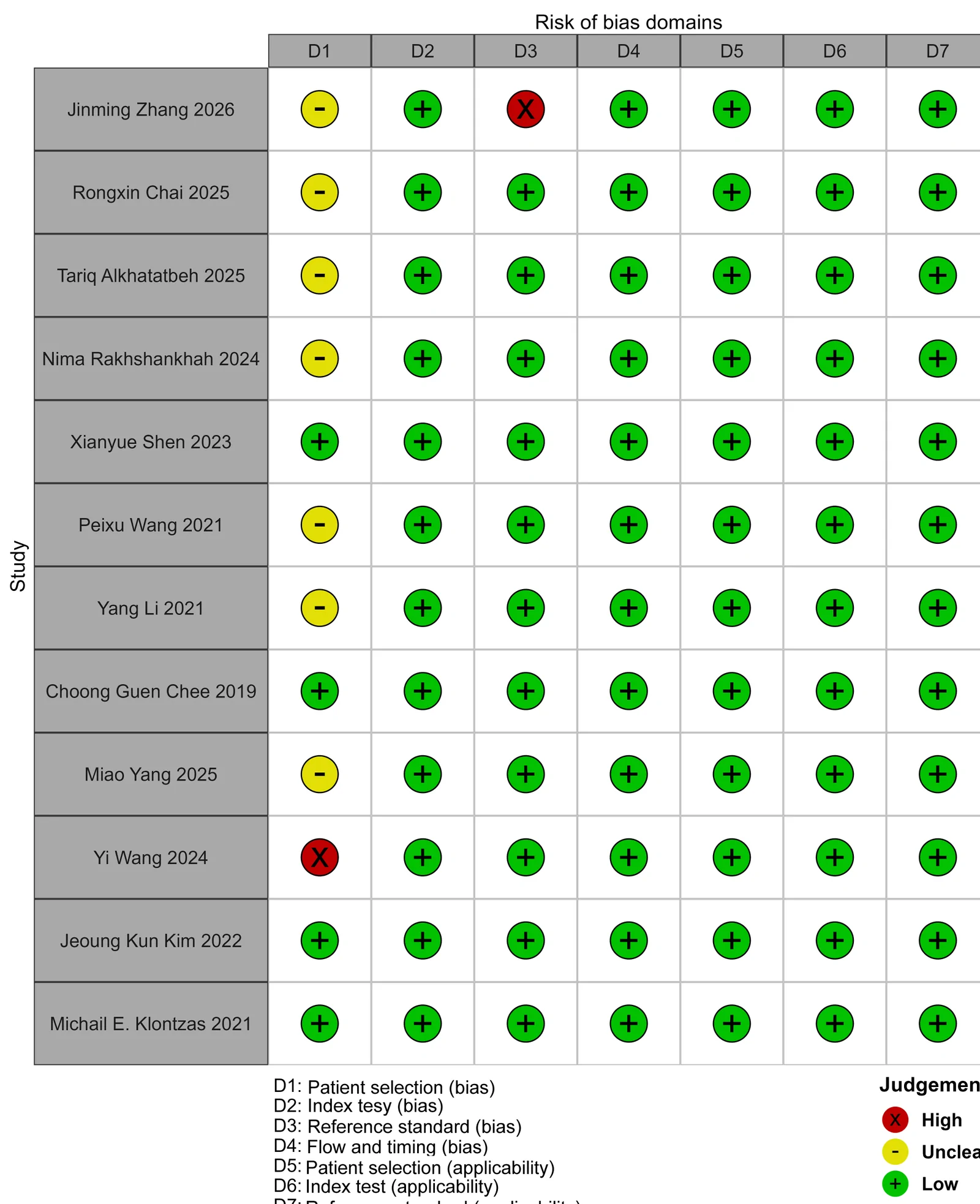
Individual risk-of-bias assessment [29-40].

The methodological quality of the 12 included studies [29-40] was assessed using the QUADAS-2 tool (Figures 2 and 3 [29-40]). Two studies were rated as having a high risk of bias in the risk-of-bias domain: 1 due to a case-control design and 1 because MRI was not explicitly used as the reference standard. Seven studies [29-33,37,38] were rated as “unclear” in the patient selection domain due to insufficient reporting of consecutive or random enrollment. The remaining domains were predominantly rated as low risk of bias. All domains in the applicability dimension were rated as low concern. Detailed results are provided in Multimedia Appendix 3.

### Meta-Analysis Results

#### Overall Diagnostic Accuracy

**Figure 4.**
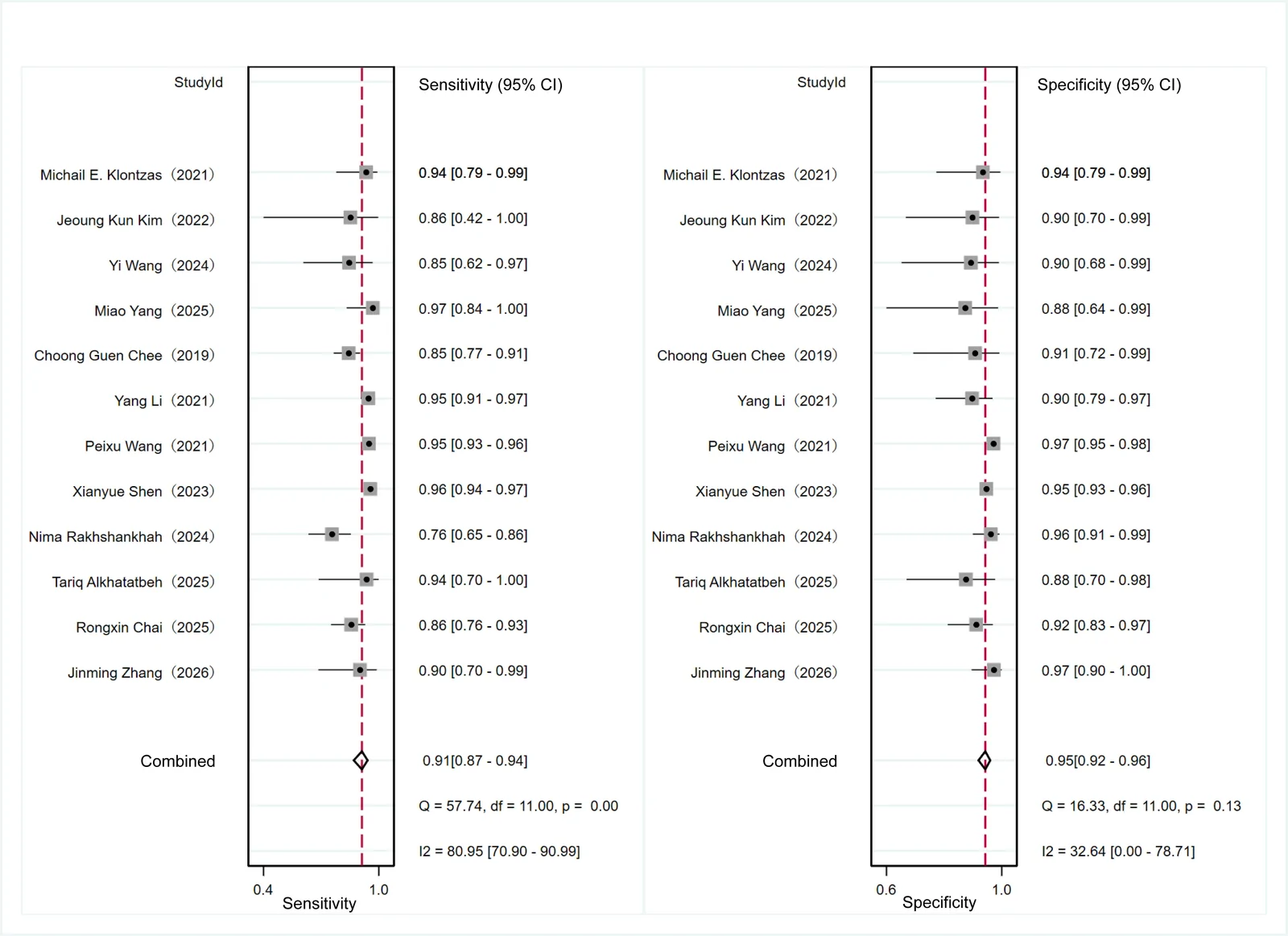
Forest plot of Sensitivity and Specificity for the AI models constructed based on medical imaging for diagnosing osteonecrosis of the femoral head [29-40].

**Figure 5.**
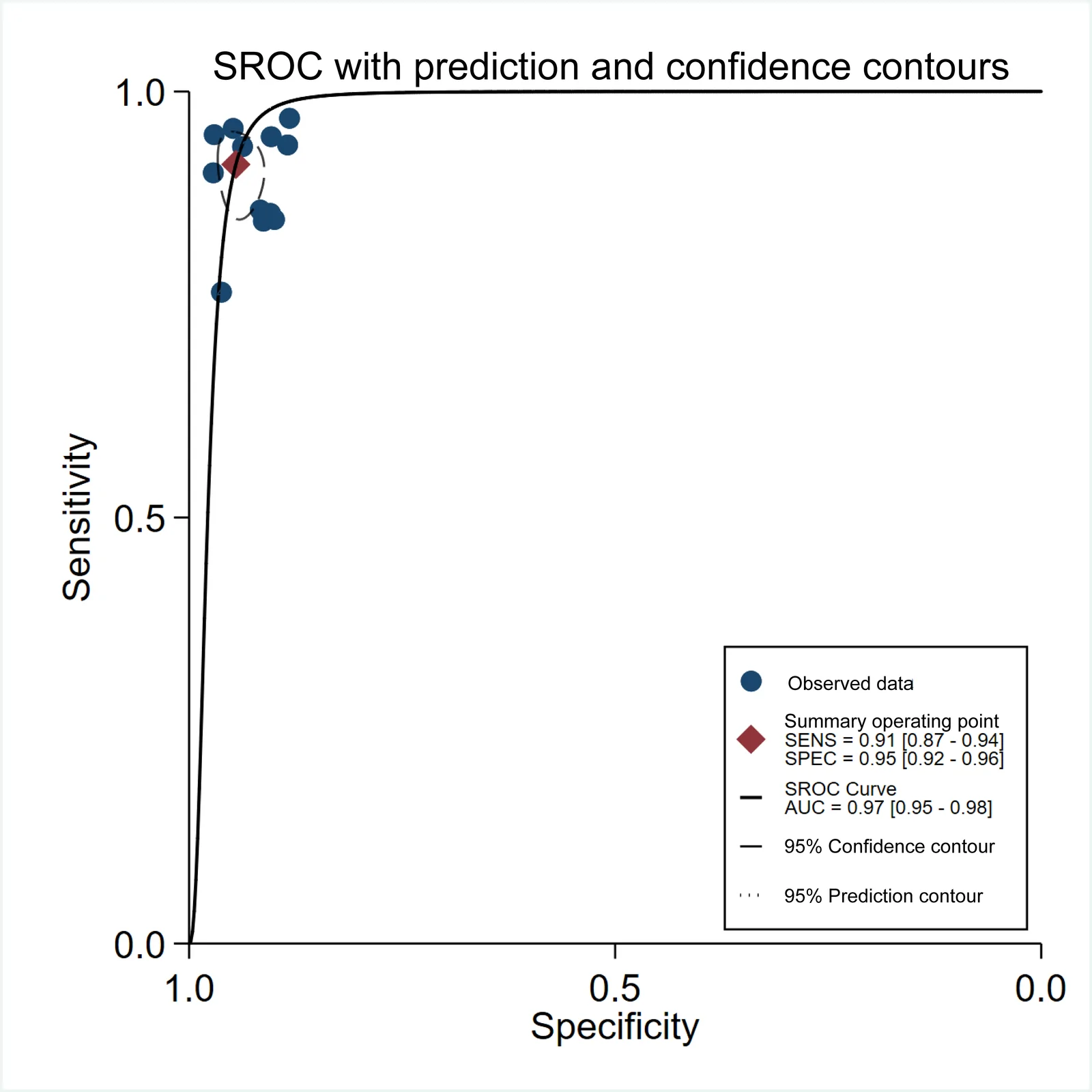
Summary receiver operating characteristic (SROC) curve for the AI models constructed based on medical imaging for diagnosing osteonecrosis of the femoral head.

**Figure 6.**
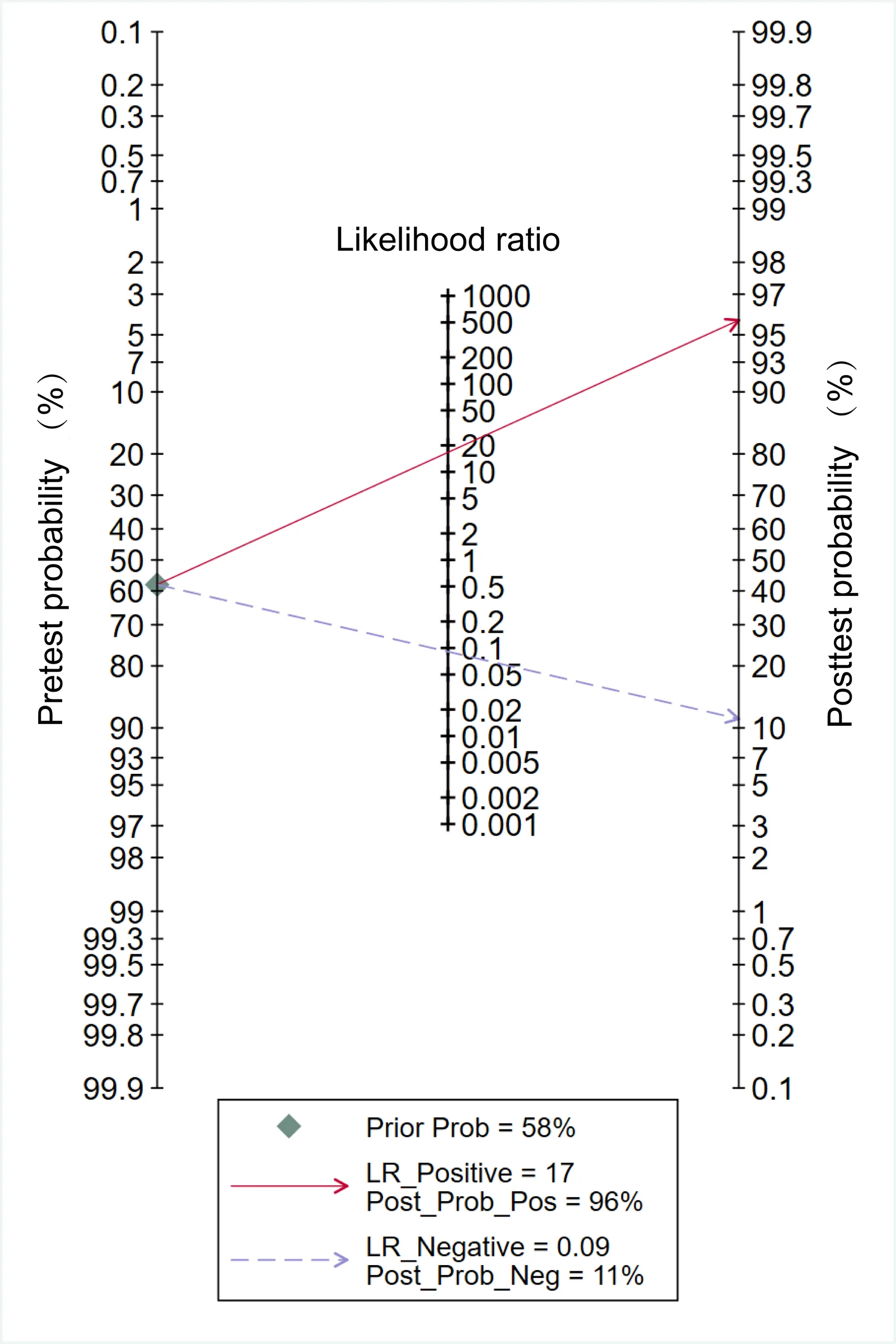
Nomogram for the AI models constructed based on medical imaging for diagnosing osteonecrosis of the femoral head.

Meta-analysis was performed using a bivariate random-effects model based on the 12 included studies [29-40]. The pooled sensitivity was 0.91 (95% CI 0.87‐0.95), pooled specificity was 0.95 (95% CI 0.93‐0.96), and the SROC AUC was 0.97 (95% CI 0.95‐0.98; Figure 4 [29-40]). The pooled PLR was 16.7 (95% CI 11.9‐23.4), pooled NLR was 0.09 (95% CI 0.06‐0.14), and pooled DOR was 184 (95% CI 101‐335; Figure 5). Between-study heterogeneity was observed (Cochran Q=7.126, *P*=.01; *I*²=72%, 95% CI 38%‐100%). Heterogeneity was substantial for sensitivity (*I*²=80.95%) but nonsignificant for specificity (*I*²=32.64%), suggesting that variability across studies was primarily driven by differences in sensitivity rather than specificity. Threshold effect analysis showed that the correlation between the logit-transformed sensitivity and specificity in the bivariate model was not significant (*r*=0.364; *P*=.65), and the Spearman correlation test also revealed no significant negative correlation (*ρ*=−0.105; *P*=.74), suggesting that no discernible threshold effect was detected. Using an overall disease prevalence of 57% as the pretest probability and incorporating a PLR of 16.7 and an NLR of 0.09, a Fagan nomogram was constructed, which showed that a positive AI prediction increased the posttest probability to 96%, while a negative prediction reduced it to 11% (Figure 6). Detailed Fagan nomogram results for all subgroups are provided in Table S1 in Multimedia Appendix 4.

#### Subgroup Analysis

##### Imaging Modality

Six studies [29,30,33,36,38,40] were included in the x-ray subgroup. The pooled sensitivity was 0.87 (95% CI 0.81‐0.92), pooled specificity was 0.94 (95% CI 0.90‐0.96), pooled PLR was 14, pooled NLR was 0.13, pooled DOR was 106 (95% CI 60‐190), and the SROC AUC was 0.97 (95% CI 0.95‐0.98; Figures S1 and S2 in Multimedia Appendix 4). Deeks test revealed no significant publication bias (Figure S3 in Multimedia Appendix 4). At a disease prevalence of 58% as the pretest probability, a PLR of 14 and an NLR of 0.13 yielded a posttest probability of 95% for a positive prediction and 16% for a negative prediction (Figure S4 in Multimedia Appendix 4).

Six studies [31,32,34,35,37,39] were included in the MRI subgroup. The pooled sensitivity was 0.95 (95% CI 0.94‐0.96), pooled specificity was 0.95 (95% CI 0.92‐0.97), pooled PLR was 20, pooled NLR was 0.05, pooled DOR was 382 (95% CI 220‐665), and the SROC AUC was 0.97 (95% CI 0.95‐0.98; Figures S5 and S6 in Multimedia Appendix 4). Deeks test revealed no significant publication bias (Figure S7 in Multimedia Appendix 4). At a disease prevalence of 56% as the pretest probability, a PLR of 20 and an NLR of 0.05 yielded a posttest probability of 96% for a positive prediction and 6% for a negative prediction (Figure S8 in Multimedia Appendix 4).

##### Diagnostic Criteria

Five studies [30,34,35,37,40] were included in the ARCO staging subgroup. The pooled sensitivity was 0.91 (95% CI 0.86‐0.95), specificity was 0.94 (95% CI 0.90‐0.96), PLR was 14.9 (95% CI 8.8‐25.3), NLR was 0.09 (95% CI 0.05‐0.16), DOR was 162 (95% CI 61‐430), and the SROC AUC was 0.97 (95% CI 0.96‐0.98; Figures S9 and S10 in Multimedia Appendix 4). Deeks test suggested potential publication bias; after correction using the trim-and-fill method, the DOR was 171.7 (95% CI 64.2‐459.3), consistent with the original estimate (Figures S11 and S12 in Multimedia Appendix 4). At a disease prevalence of 52% as the pretest probability, a PLR of 14.9 and an NLR of 0.09 corresponded to posttest probabilities of 94% for a positive prediction and 9% for a negative prediction, respectively (Figure S13 in Multimedia Appendix 4).

Seven studies [29,31-33,36,38,39] were included in the other reference standards subgroup (including Ficat staging, modified Ficat-Arlet staging, comprehensive clinical diagnosis, and MRI-based diagnosis). The pooled sensitivity was 0.92 (95% CI 0.85‐0.96), specificity was 0.93 (95% CI 0.89‐0.96), PLR was 13.8 (95% CI 8.5‐22.3), NLR was 0.09 (95% CI 0.05‐0.16), DOR was 155 (95% CI 78‐305), and the SROC AUC was 0.97 (95% CI 0.95‐0.98; Figures S14 and S15 in Multimedia Appendix 4). Deeks test showed no significant publication bias (Figure S16 in Multimedia Appendix 4). At a disease prevalence of 63% as the pretest probability, a PLR of 13.8 and an NLR of 0.09 yielded posttest probabilities of 96% and 13%, respectively (Figure S17 in Multimedia Appendix 4).

##### Disease Stage

Six studies [30,32,34-37] were included in the early-stage ONFH subgroup. The bivariate mixed-effects model yielded a pooled sensitivity of 0.93 (95% CI 0.89‐0.96), specificity of 0.94 (95% CI 0.91‐0.96), PLR of 15.7 (95% CI 9.7‐25.6), NLR of 0.08 (95% CI 0.05‐0.12), DOR of 209 (95% CI 86‐512), and an SROC AUC of 0.98 (95% CI 0.96‐0.99; Figures S18 and S19 in Multimedia Appendix 4). Deeks test showed no significant publication bias (Figure S20 in Multimedia Appendix 4). With an overall disease prevalence of 48% as the pretest probability, a PLR of 15.7 and an NLR of 0.08 yielded posttest probabilities of 94% and 6%, respectively (Figure S21 in Multimedia Appendix 4).

Six studies [29,31,33,38-40] were included in the all-stage ONFH subgroup. The bivariate mixed-effects model yielded a pooled sensitivity of 0.89 (95% CI 0.82‐0.94), specificity of 0.94 (95% CI 0.90‐0.96), PLR of 14.9 (95% CI 9.1‐24.3), NLR of 0.11 (95% CI 0.07‐0.19), DOR of 132 (95% CI 69‐251), and an SROC AUC of 0.97 (95% CI 0.95‐0.98; Figures S22 and S23 in Multimedia Appendix 4). Deeks test showed no significant publication bias (Figure S24 in Multimedia Appendix 4). With an overall disease prevalence of 65% as the pretest probability, a PLR of 14.9 and an NLR of 0.11 yielded posttest probabilities of 97% and 17%, respectively (Figure S25 in Multimedia Appendix 4).

##### Control Group Type

Eight studies [29-31,33,36,38-40] were included in the healthy controls subgroup. The bivariate mixed-effects model yielded a pooled sensitivity of 0.89 (95% CI 0.83‐0.93), specificity of 0.93 (95% CI 0.90‐0.96), PLR of 13.4 (95% CI 9‐19.9), NLR of 0.12 (95% CI 0.08‐0.18), DOR of 111 (95% CI 65‐192), and an SROC AUC of 0.96 (95% CI 0.94‐0.98; Figures S26 and S27 in Multimedia Appendix 4). Deeks test showed no significant publication bias (Figure S28 in Multimedia Appendix 4). With an overall disease prevalence of 58% as the pretest probability, a PLR of 13.4 and an NLR of 0.12 yielded posttest probabilities of 95% and 14%, respectively (Figure S29 in Multimedia Appendix 4).

Four studies [32,34,35,37] were included in the disease controls subgroup. The bivariate mixed-effects model yielded a pooled sensitivity of 0.95 (95% CI 0.94‐0.96), specificity of 0.96 (95% CI 0.94‐0.97), PLR of 21.3 (95% CI 15‐30.1), NLR of 0.05 (95% CI 0.04‐0.07), DOR of 423 (95% CI 274‐653), and an SROC AUC of 0.97 (95% CI 0.95‐0.98; Figures S30 and S31 in Multimedia Appendix 4). Deeks test showed no significant publication bias (Figure S32 in Multimedia Appendix 4). With an overall disease prevalence of 53% as the pretest probability, a PLR of 21.3 and an NLR of 0.05 yielded posttest probabilities of 96% and 5%, respectively (Figure S33 in Multimedia Appendix 4).

##### Validation Method

Seven studies [29,31,33,36-39] were included in the internal validation subgroup. The bivariate mixed-effects model yielded a pooled sensitivity of 0.92 (95% CI 0.86‐0.96), specificity of 0.95 (95% CI 0.92‐0.97), PLR of 19.5 (95% CI 11.7‐32.3), NLR of 0.08 (95% CI 0.05‐0.15), DOR of 230 (95% CI 104‐510), and an SROC AUC of 0.98 (95% CI 0.96‐0.99; Figures S34 and S35 in Multimedia Appendix 4). Deeks test suggested potential publication bias; after applying the trim-and-fill method, the corrected DOR was 201.9 (95% CI 90.8‐448.6), consistent with the original estimate (Figures S36 and S37 in Multimedia Appendix 4). With an overall disease prevalence of 64% as the pretest probability, a PLR of 19.5 and an NLR of 0.08 yielded posttest probabilities of 97% and 13%, respectively (Figure S38 in Multimedia Appendix 4).

Five studies [30,32,34,35,40] were included in the external validation subgroup. The bivariate mixed-effects model yielded a pooled sensitivity of 0.91 (95% CI 0.85‐0.95), specificity of 0.93 (95% CI 0.89‐0.95), PLR of 12.6 (95% CI 7.8‐20.6), NLR of 0.10 (95% CI 0.06‐0.17), DOR of 129 (95% CI 51‐329), and an SROC AUC of 0.96 (95% CI 0.94‐0.98; Figures S39 and S40 in Multimedia Appendix 4). Deeks test showed no significant publication bias (Figure S41 in Multimedia Appendix 4). With an overall disease prevalence of 51% as the pretest probability, a PLR of 12.6 and an NLR of 0.10 yielded posttest probabilities of 93% and 9%, respectively (Figure S42 in Multimedia Appendix 4).

##### Center Type

Six studies [29,31,36-39] were included in the single-center subgroup. The bivariate mixed-effects model yielded a pooled sensitivity of 0.95 (95% CI 0.93‐0.96), specificity of 0.95 (95% CI 0.91‐0.97), PLR of 18.2 (95% CI 10.3‐32.4), NLR of 0.06 (95% CI 0.04‐0.08), DOR of 318 (95% CI 154‐654), and an SROC AUC of 0.96 (95% CI 0.94‐0.97; Figures S43 and S44 in Multimedia Appendix 4). Deeks test suggested potential publication bias; after applying the trim-and-fill method, the corrected DOR was 270.6 (95% CI 127.4‐575.1), consistent with the original estimate (Figures S45 and S46 in Multimedia Appendix 4). With an overall disease prevalence of 68% as the pretest probability, a PLR of 18.2 and an NLR of 0.06 yielded posttest probabilities of 97% and 11%, respectively (Figure S47 in Multimedia Appendix 4).

Six studies [30,32-35,40] were included in the multicenter subgroup. The bivariate mixed-effects model yielded a pooled sensitivity of 0.89 (95% CI 0.82‐0.94), specificity of 0.94 (95% CI 0.92‐0.96), PLR of 15.5 (95% CI 10.7‐22.4), NLR of 0.11 (95% CI 0.07‐0.20), DOR of 135 (95% CI 63‐291), and an SROC AUC of 0.95 (95% CI 0.93‐0.96; Figures S48 and S49 in Multimedia Appendix 4). Deeks test suggested potential publication bias; after applying the trim-and-fill method, the corrected DOR was 115 (95% CI 45‐293), consistent with the original estimate (Figures S50 and S51 in Multimedia Appendix 4). With an overall disease prevalence of 50% as the pretest probability, a PLR of 15.5 and an NLR of 0.11 yielded posttest probabilities of 94% and 10%, respectively (Figure S52 in Multimedia Appendix 4).

##### Model Type

Nine studies [29,30,32,33,35-38,40] were included in the DL subgroup. The bivariate mixed-effects model yielded a pooled sensitivity of 0.91 (95% CI 0.87‐0.95), specificity of 0.95 (95% CI 0.93‐0.96), PLR of 18 (95% CI 12.8‐25.4), NLR of 0.09 (95% CI 0.06‐0.14), DOR of 201 (95% CI 105‐386), and an SROC AUC of 0.97 (95% CI 0.95‐0.98; Figures S53 and S54 in Multimedia Appendix 4). Deeks test suggested potential publication bias; after applying the trim-and-fill method, the corrected DOR was 179.8 (95% CI 91‐355.3), consistent with the original estimate (Figures S55 and S56 in Multimedia Appendix 4). With an overall disease prevalence of 57% as the pretest probability, a PLR of 18.0 and an NLR of 0.09 yielded posttest probabilities of 96% and 11%, respectively (Figure S57 in Multimedia Appendix 4).

The ML subgroup comprised only 3 studies [31,34,39] and was not subjected to quantitative pooling; their individual sensitivities were 0.94, 0.85, and 0.94, with specificities of 0.89, 0.90, and 0.94, respectively.

### Meta-Regression

Univariate meta-regression showed that imaging modality had a significant effect on log DOR (β=1.36, 95% CI 0.50‐2.21; *P*=.005), explaining 92.1% of the between-study heterogeneity (adjusted *R*²=92.07%). The validation method did not reach statistical significance (β=−0.44, 95% CI −1.84 to 0.96; *P*=.50). In the multivariable meta-regression model incorporating both imaging modality and validation method, the overall model was statistically significant (*F*_2,9_=8.03; *P*=.01). The independent contribution of imaging modality remained significant (β=1.47, 95% CI 0.62‐2.31; *P*=.003), whereas the contribution of validation method was not significant (β=−.50, 95% CI −1.32 to –0.31; *P*=.20). Together, the 2 covariates explained 95.3% of the between-study heterogeneity (*R*²=95.3%).

### Sensitivity Analysis

In the leave-one-out sensitivity analysis, the pooled DOR ranged from 137.1 to 190.6 across iterations, with the 95% CI of all estimates overlapping with the overall pooled DOR (184, 95% CI 101‐335). This indicated that no single study exerted a disproportionate influence on the pooled estimate, confirming the robustness of the meta-analysis results.

## Discussion

### Principal Findings and Clinical Implications

Early diagnosis is critical to the prognosis of ONFH. Without intervention, the rate of femoral head collapse within three years can reach 76% [41], and 50%‐75% of patients with advanced-stage disease ultimately require THA [42,43]. Data on hip-preserving outcomes stratified by disease stage and collapse severity further substantiate the clinical value of timely diagnosis. Reported hip preservation success rates were 97.67% for ARCO stage I–II, 91.53% for stage IIIA, and 70.59% for stage IIIB. Among patients with femoral head collapse ≤2 mm, the success rate reached 94.12%, compared with only 70.59% in those with collapse >2 mm [44]. In summary, the timing of diagnosis directly determines the clinical outcome of hip-preserving treatment in patients with ONFH.

MRI is currently the imaging modality of choice for early ONFH [45], as it can precisely identify bone marrow edema and subchondral trabecular microarchitectural injury, 2 characteristic imaging features of early-stage ONFH lesions. A systematic review by Parikh et al [46] reported a pooled sensitivity of 0.91 (95% CI 0.87‐0.94) and a pooled specificity of 0.96 (95% CI 0.87‐0.99) for MRI-based diagnosis of ONFH. In contrast, x-ray yielded a sensitivity of only 0.50 (95% CI 0.33‐0.68) and a specificity of 0.61 (95% CI 0.26‐0.87), underscoring the substantial limitations of x-ray in detecting early-stage lesions. Furthermore, the ARCO international clinical practice guidelines explicitly state that MRI can not only determine whether hip pain is attributable to ONFH but also confirm that bone marrow edema, rather than joint effusion, accounts for the patient’s hip pain, thereby providing critical imaging evidence to support individualized clinical decision-making in ONFH [47].

With the rapid advancement of AI, AI-assisted imaging diagnosis has emerged as a novel approach to improving the efficiency of early ONFH screening. The present meta-analysis validated the diagnostic performance of imaging-based AI models for ONFH, yielding a pooled sensitivity of 0.91 (95% CI 0.87‐0.95), a pooled specificity of 0.95 (95% CI 0.93‐0.96), and an SROC AUC of 0.97 (95% CI 0.95‐0.98). Collectively, these findings confirm that AI models constructed from medical imaging demonstrate high overall diagnostic accuracy for ONFH, are capable of compensating for the clinical limitations of conventional manual image interpretation, and have the potential to serve as a standardized adjunctive tool for early ONFH screening and assisted diagnosis in clinical practice.

In this study, MRI-based AI models achieved a pooled sensitivity of 0.95, a diagnostic performance comparable to that of independent interpretation by experienced senior clinicians. More importantly, AI models offer the advantage of automated image analysis. They can generate preliminary reading results in real time, accurately annotate suspicious lesion regions, automatically prioritize high-risk cases for expert review, and produce standardized, reproducible quantitative assessments. This workflow enables senior clinicians to delegate the repetitive review of low-risk negative cases to AI, allowing them to focus on diagnostically challenging ONFH cases with atypical imaging presentations. Across 11 head-to-head comparative studies between AI and clinicians, the diagnostic performance of the AI model surpassed that of junior and intermediate-level clinicians in the majority of these comparisons. For less experienced clinicians, AI-generated lesion localization annotations and diagnostic probability scores can thus assist in identifying subtle early ONFH features that are easily missed on imaging, narrowing the diagnostic gap between novices and senior experts and facilitating the standardized improvement of clinical diagnostic proficiency. Given the limited availability of MRI equipment and the uneven distribution of imaging resources in primary care settings, x-ray remains the most commonly used first-line imaging modality for hip disease screening at the community level. In this study, x-ray-based AI models achieved a sensitivity of 0.87 and a specificity of 0.94, representing a substantial improvement over the reported sensitivity of 0.50 for manual x-ray interpretation. In the early-stage ONFH subgroup, the pooled sensitivity of AI models reached 0.93, indicating that the superior ability of AI to detect early ONFH lesions does not solely rely on the superior soft-tissue contrast of MRI. Even on x-ray images, where the information content is limited and soft-tissue resolution is relatively poor, AI algorithms can extract subtle trabecular bone abnormalities that are imperceptible to the human eye, thereby providing primary care clinicians with a reliable diagnostic reference for early ONFH screening.

The Fagan nomogram analysis provided quantitative evidence supporting the clinical utility of the AI models from a clinical decision-making perspective. Using an overall ONFH prevalence of 57% in the study population as the baseline pretest probability, a positive AI prediction raised the posttest probability of ONFH to 96%, while a negative AI prediction reduced it to 11%. An AI-negative result can effectively rule out ONFH, helping clinicians avoid unnecessary high-cost MRI examinations and reducing patients’ financial burden. An AI-positive result, by contrast, identifies patients at high risk of ONFH who should be prioritized for confirmatory MRI evaluation.

In the model-type subgroup analysis exploring differences in diagnostic performance across AI algorithms, DL models yielded a pooled DOR of 201 (95% CI 105‐386) for ONFH diagnosis. Only 3 included studies [31,34,39] used traditional ML algorithms, precluding quantitative synthesis owing to the limited number of studies and total sample size; the individual sensitivities of these 3 models were 0.94, 0.85, and 0.94, with corresponding specificities of 0.89, 0.90, and 0.94. The two approaches differ fundamentally in how they utilize imaging information: DL automatically extracts subtle textural and morphological features, whereas traditional ML relies on handcrafted radiomic features whose performance is constrained by the quality of the prior feature engineering. However, it remains difficult to disentangle whether the superior performance of a model reflects genuine architectural advantages or is attributable to other factors such as data scale, annotation quality, and validation strategy. The models included in this review encompassed diverse architectures, including general-purpose classification networks such as ResNet (Microsoft Research) and DenseNet (Cornell University) for qualitative ONFH diagnosis, object detection networks such as YOLO (Ultralytics) for localizing suspicious lesions on x-ray images, and segmentation networks such as U-Net (University of Freiburg) and PointRend (Meta AI) for precise quantification of necrotic regions on MRI. Given that these architectures address distinct clinical tasks, cross-comparison using a single accuracy metric is of limited value. Future studies should conduct standardized benchmarking of different architectures on unified reference datasets and develop task-specific evaluation frameworks tailored to detection and segmentation objectives.

Furthermore, the DOR in the disease controls subgroup was higher than that in the healthy controls subgroup. This phenomenon may be attributable to differences in the distribution of imaging features between the comparator conditions and ONFH. The visual discriminability of certain conditions (eg, fractures, postoperative changes, and tumors) from ONFH may even exceed that between ONFH and normal bone, rendering these cases more readily excluded by the model. In addition, the composition of disease severity within the comparator group (eg, the inclusion of advanced-stage cases) and differences in the design of multiclass classification tasks may also inflate the DOR estimate. Moreover, the definition of healthy controls was insufficiently rigorous in some studies, further complicating the intergroup comparisons. These findings warrant validation in future studies using more rigorously designed differential diagnosis cohorts.

### Methodological Quality and Sources of Heterogeneity

With respect to methodological quality, the majority of the included studies were rated as having a low or unclear risk of bias on the QUADAS-2 assessment, and all studies were classified as having low concern regarding applicability, indicating that the overall methodological quality of the included studies was acceptable. The risk of bias in the domain of patient selection constituted the primary methodological shortcoming, one that is commonly observed in the field of AI-based medical imaging, as most studies used retrospective designs with insufficient reporting of enrollment protocols, making it difficult to ascertain whether consecutive enrollment was achieved. The possibility that selective enrollment may have contributed to an overestimation of diagnostic performance cannot therefore be excluded.

With regard to publication bias, Deeks test revealed evidence of significant publication bias in the internal validation, single-center, and DL subgroups. However, after applying the trim-and-fill correction, the 95% CIs of the corrected DORs in all subgroups overlapped with those of the original estimates, suggesting that the influence of such publication bias on the principal diagnostic conclusions of this study is limited. It should be noted that Deeks test has low statistical power when the number of studies is small, and AI-based diagnosis is a rapidly expanding field in which negative results or models with suboptimal performance are less likely to be reported and published. The pooled effect estimates reported in this study should therefore be interpreted with caution, as some degree of positive-outcome reporting bias may exist.

The present meta-analysis revealed substantial between-study heterogeneity (*I*²=72%). This degree of heterogeneity is not uncommon in the field of AI-based medical imaging, and similar levels of heterogeneity have been reported in comparable systematic reviews [48,49]. This high degree of heterogeneity likely reflects multiple sources of variation. Imaging modality represents an important contributing factor, as the varying amount of information conveyed by different imaging techniques directly affects diagnostic performance. The early-stage ONFH subgroup exhibited relatively lower heterogeneity, suggesting that restricting the study population to early-stage disease may reduce case-mix variation. Validation strategy further contributed to between-study variability: although both the internal and external validation subgroups showed high heterogeneity, external validation yielded more conservative pooled estimates that more closely approximate model performance in real-world clinical settings. Single-center studies exhibited lower heterogeneity, whereas multi-center studies showed high heterogeneity, indicating that differences in imaging protocols, equipment, and patient populations across centers constitute an important source of variation. The DL subgroup showed high heterogeneity, reflecting the diversity in model architectures, training strategies, and input features. Notably, the stratified analyses revealed that heterogeneity was concentrated predominantly in the sensitivity dimension, with low heterogeneity in the specificity dimension, suggesting that AI models demonstrate good cross-study consistency in excluding non-ONFH cases. Given the high heterogeneity observed, the pooled estimates reported in this study should be interpreted as exploratory rather than definitive, and caution should be exercised when extrapolating them to specific clinical contexts.

### Advantages and Limitations

This systematic review and meta-analysis on imaging-based AI models for ONFH diagnosis provided an evidence-based foundation for the field. The core clinical value of this study lies in demonstrating that AI models can extend diagnostic capability approaching that of senior clinicians from MRI to the first-line x-ray screening setting in primary care. At the same time, they improve interpretation consistency through automated workflows and structured second-reader functions, thereby enhancing clinician efficiency.

Nevertheless, several methodological and clinical limitations should be acknowledged. Owing to the limited number of published studies in this domain, only 12 eligible original studies were included, with some subgroup analyses comprising as few as 2‐3 studies, yielding insufficient statistical power; the corresponding subgroup findings should therefore be considered as exploratory leads for future investigation. First, the included studies were predominantly retrospective in design with enrichment-based sampling strategies, which amplify imaging differences between cases and controls. Second, only 5 studies [30,32,34,35,40] used external validation, and their DOR was substantially lower than that of internally validated studies, confirming a marked attenuation in model generalizability and suggesting that the current pooled estimates are likely optimistic. Third, the included studies were geographically concentrated in East Asia (China: n=8 [29-32,34,35,37,38]; South Korea: n=2 [36,40]); given regional differences in ONFH etiology, imaging equipment, and acquisition parameters, the generalizability of the findings to other populations remains to be validated. Finally, in the comparative studies between AI and clinicians, considerable heterogeneity in clinician seniority, comparison metrics, and statistical methods precluded quantitative synthesis; the majority of these studies did not use a noninferiority design, leaving unanswered the question of whether AI is noninferior to expert clinicians, a central issue for clinical translation.

### Future Directions

Translating AI-assisted ONFH diagnosis from research into clinical practice requires advancements in several key areas. First, prospective, multicenter clinical implementation studies are needed to embed AI systems into real-world imaging workflows, evaluate their performance stability across different equipment and acquisition parameters, and delineate their scope and boundaries of applicability. Second, multimodal models integrating x-ray, MRI, and clinical text data should be developed to enhance diagnostic robustness, with large language models used to generate structured reports whose interpretive logic can be verified by clinicians. Third, building on QUADAS-2 and incorporating AI-specific reporting standards such as Transparent Reporting of a Multivariable Prediction Model for Individual Prognosis or Diagnosis–Artificial Intelligence (TRIPOD-AI) and Checklist for Artificial Intelligence in Medical Imaging (CLAIM) [50,51], standardized and publicly available ONFH imaging benchmark datasets should be established to provide a unified comparison platform for different algorithms. In parallel, external validation across different regions, ethnicities, and equipment conditions is essential to confirm model generalizability in real-world settings. Finally, randomized controlled trials are needed to prospectively evaluate the impact of AI-assisted diagnosis on clinical endpoints such as early ONFH detection rates, hip preservation rates, and long-term joint replacement rates, thereby providing high-level evidence to support clinical adoption. Systematic progress along these directions is essential for translating AI-assisted ONFH diagnosis from research into routine clinical practice.

## Conclusions

This systematic review and meta-analysis evaluated the diagnostic accuracy of medical imaging-based AI models for ONFH. The pooled results indicate that AI models demonstrate favorable diagnostic accuracy. However, owing to substantial between-study heterogeneity arising from variations in imaging modality, reference standard, and validation strategy across the included studies, the exclusively retrospective design of all included studies, and the potential risk of selective reporting bias, the robustness and generalizability of the current evidence remain uncertain, and the performance metrics should be interpreted with caution. Nevertheless, the clinical potential of AI-assisted diagnostic tools merits attention, as they can extend standardized, highly reproducible imaging interpretation to early screening and primary care settings and hold promise for shifting the diagnostic window to the precollapse stage, thereby securing a critical window for hip-preserving treatment. Future research should standardize the diagnostic and staging criteria for ONFH, adopt multicenter prospective designs with rigorous external validation, and advance research on model interpretability to confirm the robustness, transportability, and clinical applicability of AI models in ONFH diagnosis.

## Supplementary material

10.2196/95648 Multimedia Appendix 1 Literature search strategy.

10.2196/95648 Multimedia Appendix 2 Characteristics of the included studies.

10.2196/95648 Multimedia Appendix 3 Quality assessment of diagnostic accuracy studies assessment process for included studies.

10.2196/95648 Multimedia Appendix 4 Subgroup analyses and Fagan nomogram results.

10.2196/95648 Checklist 1 PRISMA-DTA checklist.

10.2196/95648 Checklist 2 PRISMA-DTA for Abstracts checklist.

## Abbreviations

ARCO: Association Research Circulation Osseous
AUC: area under the receiver operating characteristic curve
CLAIM: Checklist for Artificial Intelligence in Medical Imaging
DL: deep learning
DOR: diagnostic odds ratio
FN: false negative
FP: false positive
ML: machine learning
MRI: magnetic resonance imaging
NLR: negative likelihood ratio
NPV: negative predictive value
ONFH: osteonecrosis of the femoral head
PLR: positive likelihood ratio
PPV: positive predictive value
PRISMA-DTA: Preferred Reporting Items for Systematic Reviews and Meta-Analyses of Diagnostic Test Accuracy
PRMS-Net: Progressive Residual Multi-Class Support Vector Machine-Net
PROSPERO: International Prospective Register of Systematic Reviews
QUADAS-2: Quality Assessment of Diagnostic Accuracy Studies-2
Robvis: Risk-of-Bias Visualization
SROC: summary receiver operating characteristic
THA: total hip arthroplasty
TN: true negative
TP: true positive
TRIPOD-AI: Transparent Reporting of a Multivariable Prediction Model for Individual Prognosis or Diagnosis–Artificial Intelligence
