# Supplementary material for: Diagnostic Accuracy of Medical Imaging–Based Artificial Intelligence for Osteonecrosis of the Femoral Head: Systematic Review and Meta-Analysis
Source: J Med Internet Res. 2026 Aug 20;28:e95648. doi: 10.2196/95648 (PMC13492484; doi:10.2196/95648)
Supplement: Supplementary file 2 [file jmir-v28-e95648-s006.pdf]

PRISMA-DTA for Abstracts Checklist

Section/topic	#	PRISMA-DTA for Abstracts Checklist item	Reported on page #
**TITLE and PURPOSE**			
Title	1	Identify the report as a systematic review (+/- meta-analysis) of diagnostic test accuracy (DTA) studies.	1
Objectives	2	Indicate the research question, including components such as participants, index test, and target conditions.	1
**METHODS**			
Eligibility criteria	3	Include study characteristics used as criteria for eligibility.	1
Information sources	4	List the key databases searched and the search dates.	1
Risk of bias & applicability	5	Indicate the methods of assessing risk of bias and applicability.	1
Synthesis of results	A1	Indicate the methods for the data synthesis.	1-2
**RESULTS**			
Included studies	6	Indicate the number and type of included studies and the participants and relevant characteristics of the studies (including the reference standard).	2
Synthesis of results	7	Include the results for the analysis of diagnostic accuracy, preferably indicating the number of studies and participants. Describe test accuracy including variability; if meta-analysis was done, include summary results and confidence intervals.	2
**DISCUSSION**			
Strengths and limitations	9	Provide a brief summary of the strengths and limitations of the evidence	2
Interpretation	10	Provide a general interpretation of the results and the important implications.	2
**OTHER**			
Funding	11	Indicate the primary source of funding for the review.	-
Registration	12	Provide the registration number and the registry name	1

Adapted From: McInnes MDF, Moher D, Thombs BD, McGrath TA, Bossuyt PM, The PRISMA-DTA Group (2018). Preferred Reporting Items for a Systematic Review and Meta-analysis of Diagnostic Test Accuracy Studies: The PRISMA-DTA Statement. JAMA. 2018 Jan 23;319(4):388-396. doi: 10.1001/jama.2017.19163.
