# Supplementary material for: Diagnostic Accuracy of Medical Imaging–Based Artificial Intelligence for Osteonecrosis of the Femoral Head: Systematic Review and Meta-Analysis
Source: J Med Internet Res. 2026 Aug 20;28:e95648. doi: 10.2196/95648 (PMC13492484; doi:10.2196/95648)
Supplement: Supplementary file 3 [file jmir-v28-e95648-s001.docx]

1. **Pubmed**

Date Run: 2026/3/8

| Search number | Query | Results |
| --- | --- | --- |
| #1 | "Femur Head Necrosis"[Mesh] | 9328 |
| #2 | ((((((((Femoral Head Necrosis) OR (Osteonecrosis of the femoral head)) OR (Avascular necrosis of the femoral head)) OR (Aseptic necrosis of the femoral head)) OR (Ischemic necrosis of the femoral head)) OR (ONFH)) OR (ANFH)) OR (Femoral Head Osteonecrosis)) OR (Hip Osteonecrosis) | 15980 |
| #3 | (((((((((Femoral Head Necrosis) OR (Osteonecrosis of the femoral head)) OR (Avascular necrosis of the femoral head)) OR (Aseptic necrosis of the femoral head)) OR (Ischemic necrosis of the femoral head)) OR (ONFH)) OR (ANFH)) OR (Femoral Head Osteonecrosis)) OR (Hip Osteonecrosis)) OR ("Femur Head Necrosis"[Mesh]) | 15980 |
| #4 | "Machine Learning"[Mesh] | 117122 |
| #5 | "Deep Learning"[Mesh] | 39656 |
| #6 | ((((((((((((((((((((((((machine learning) OR (Transfer Learning)) OR (Deep learning)) OR (Ensemble Learning)) OR (artificial intelligence)) OR (random forest)) OR (neural network)) OR (K-Nearest Neighbor)) OR (CNN)) OR (Support vector machine)) OR (SVM)) OR (Gradient Boosting)) OR (Machine Nomogram)) OR (XGBoost)) OR (Adaboost)) OR (Decision tree)) OR (ResNet)) OR (AlexNet)) OR (VGGNet)) OR (GoogLeNet)) OR (LSTMs)) OR (Naive Bayesian)) OR (Multilayer perceptron)) OR (Bayesian network)) OR (Radiomic) | 668600 |
| #6 | ((((((((((((((((((((((((((machine learning) OR (Transfer Learning)) OR (Deep learning)) OR (Ensemble Learning)) OR (artificial intelligence)) OR (random forest)) OR (neural network)) OR (K-Nearest Neighbor)) OR (CNN)) OR (Support vector machine)) OR (SVM)) OR (Gradient Boosting)) OR (Machine Nomogram)) OR (XGBoost)) OR (Adaboost)) OR (Decision tree)) OR (ResNet)) OR (AlexNet)) OR (VGGNet)) OR (GoogLeNet)) OR (LSTMs)) OR (Naive Bayesian)) OR (Multilayer perceptron)) OR (Bayesian network)) OR (Radiomic)) OR ("Machine Learning"[Mesh])) OR ("Deep Learning"[Mesh]) | 668600 |
| #7 | (((((((((((((((((((((((((((machine learning) OR (Transfer Learning)) OR (Deep learning)) OR (Ensemble Learning)) OR (artificial intelligence)) OR (random forest)) OR (neural network)) OR (K-Nearest Neighbor)) OR (CNN)) OR (Support vector machine)) OR (SVM)) OR (Gradient Boosting)) OR (Machine Nomogram)) OR (XGBoost)) OR (Adaboost)) OR (Decision tree)) OR (ResNet)) OR (AlexNet)) OR (VGGNet)) OR (GoogLeNet)) OR (LSTMs)) OR (Naive Bayesian)) OR (Multilayer perceptron)) OR (Bayesian network)) OR (Radiomic)) OR ("Machine Learning"[Mesh])) OR ("Deep Learning"[Mesh])) AND ((((((((((Femoral Head Necrosis) OR (Osteonecrosis of the femoral head)) OR (Avascular necrosis of the femoral head)) OR (Aseptic necrosis of the femoral head)) OR (Ischemic necrosis of the femoral head)) OR (ONFH)) OR (ANFH)) OR (Femoral Head Osteonecrosis)) OR (Hip Osteonecrosis)) OR ("Femur Head Necrosis"[Mesh])) | 148 |

2. **Cochrane**

Date Run: 2026/3/8

| Search number | Query | Results |
| --- | --- | --- |
| #1 | (Femoral Head Necrosis)t.ab,kw OR (Osteonecrosis of the femoral head) ti.ab,kw OR (Avascular necrosis of the femoral head) ti.ab,kw OR (Aseptc necrosis of the femoral head):ti,ab,kw OR (Ischemic necrosis of the femoral head):ti,ab,kw | 679 |
| #2 | ONFH):ti,ab,kw OR (ANFH):ti,ab.kw OR (Femoral Head Osteonecrosis).t,ab,kw OR (Hip Osteonecrosis):ti,ab,kw | 547 |
| #3 | MeSH descriptor: [Femur Head Necrosis] explode all trees | 200 |
| #4 | #1 OR #2 OR #3 | 916 |
| #5 | MeSH descriptor: [Machine Learning] explode all trees | 1266 |
| #6 | MeSH descriptor: [Deep Learning] explode all trees | 409 |
| #8 | (machine learning):ti,ab,kw OR (TransferLearning):ti,ab,kw OR (Deep learning):ti,ab,kw OR(Ensemble Learning):ti,ab,kw OR (artificialintelligence):ti,ab,kw | 10943 |
| #9 | (random forest):ti,ab,kw OR (neural network):ti,ab,kwOR (K-Nearest Neighbor):ti,ab,kw OR (CNN):ti,ab,kwOR (Support vector machine):ti,ab,kw | 7402 |
| #10 | (SVM):ti,ab,kw OR (Gradient Boosting):ti,ab,kw OR (MachineNomogram):ti,ab,kw OR (XGBoost):ti,ab,kw OR(Adaboost):ti,ab,kw | 825 |
| #11 | (Decision tree):ti,ab,kw OR (ResNet):ti,ab,kw OR(AlexNet):ti,ab,kw OR (VGGNet):ti,ab,kw OR(GoogLeNet):ti,ab,kw | 1442 |
| #12 | (LSTMs):ti,ab,kw OR (Naive Bayesian):ti,ab,kw OR (Multilayerperceptron):ti,ab,kw OR (Bayesian network):ti,ab,kw OR(Radiomic):ti,ab,kw | 1493 |
| #13 | #6 OR #7 OR #8 OR #9 OR #10 OR #11 OR #12 | 18587 |
| #14 | #4 AND #12 | 6 |

3. **Embase**

Date Run:2026/3/8

| Search number | Query | Results |
| --- | --- | --- |
| #1 | 'Femoral Head Necrosis'/exp | 8868 |
| #2 | 'femoral head necrosis':ab,ti OR 'osteonecrosis of the femoral head':ab,ti OR 'avascular necrosis of the femoral head':ab,ti OR 'aseptic necrosis of the femoral head':ab,ti OR 'ischemic necrosis of the femoral head':ab,ti OR anfh:ab,ti OR onfh:ab,ti OR 'femoral head osteonecrosis':ab,ti OR 'hip osteonecrosis':ab,ti | 8098 |
| #3 | 'machine learning':ab,ti OR 'transfer learning':ab,ti OR 'deep learning':ab,ti OR 'ensemble learning':ab,ti OR 'artificial intelligence':ab,ti OR 'random forest':ab,ti OR 'neural network':ab,ti OR 'k-nearest neighbor':ab,ti OR cnn:ab,ti OR 'support vector machine':ab,ti OR svm:ab,ti OR 'gradient boosting':ab,ti OR 'machine nomogram':ab,ti OR xgboost:ab,ti OR adaboost:ab,ti OR 'decision tree':ab,ti OR resnet:ab,ti OR alexnet:ab,ti OR vggnet:ab,ti OR googlenet:ab,ti OR lstms:ab,ti OR 'naive bayesian':ab,ti OR 'multilayer perceptron':ab,ti OR 'bayesian network':ab,ti OR radiomic:ab,ti | 500164 |
| #4 | 'machine learning'/exp | 716024 |
| #5 | 'deep learning'/exp | 109594 |
| #6 | #1 OR #2 | 11881 |
| #7 | #3 OR #4 OR #5 | 856977 |
| #8 | #6 AND #7 | 168 |

4. **Web of science**

Date Run: 2026/3/8

| Search number | Query | Results |
| --- | --- | --- |
| #1 | Femoral Head Necrosis (Topic) or Osteonecrosis of the femoral head (All Fields) or Avascular necrosis of the femoral head (All Fields) or Aseptic necrosis of the femoral head (All Fields) or Ischemic necrosis of the femoral head (All Fields) or ONFH (All Fields) or ANFH (All Fields) or Femoral Head Osteonecrosis (All Fields) or Hip Osteonecrosis (All Fields) | 6243 |
| #2 | machine learning (Topic) or Transfer Learning (Topic) or Deep learning (Topic) or Ensemble Learning (Topic) or artificial intelligence (Topic) or random forest (Topic) or neural network (Topic) or neural networks (Topic) or K-Nearest Neighbor (Topic) or CNN (Topic) or Support vector machine (Topic) or SVM (Topic) or XGBoost (Topic) or Adaboost (Topic) or Decision tree (Topic) or ResNet-50 (Topic) or ResNet (Topic) or AlexNet (Topic) or VGGNet (Topic) or GoogLeNet (Topic) or LSTMs (Topic) or Naive Bayesian (Topic) or Multilayer perceptron (Topic) or Bayesian network (Topic) or Radiomics (Topic) or Radiomic (Topic) | 1405692 |
| #3 | #1 AND #2 | 243 |
