# Supplementary material for: Diagnostic Accuracy of Medical Imaging–Based Artificial Intelligence for Osteonecrosis of the Femoral Head: Systematic Review and Meta-Analysis
Source: J Med Internet Res. 2026 Aug 20;28:e95648. doi: 10.2196/95648 (PMC13492484; doi:10.2196/95648)
Supplement: Supplementary file 4 [file jmir-v28-e95648-s004.docx]

**Supplementary Figure 1.** Forest plot of sensitivity and specificity for the AI models constructed based on X-ray for diagnosing ONFH.

**Supplementary Figure 2.** SROC curve for the AI models constructed based on X-ray for diagnosing ONFH.

**Supplementary Figure 3.** Deeks' test for the AI models constructed based on X-ray for diagnosing ONFH.

**Supplementary Figure 4.** Fagan's nomogram for the AI models constructed based on X-ray for diagnosing ONFH.

**Supplementary Figure 5.** Forest plot of sensitivity and specificity for the AI models constructed based on MRI for diagnosing ONFH.

**Supplementary Figure 6.** SROC curve for the AI models constructed based on MRI for diagnosing ONFH.

**Supplementary Figure 7.** Deeks' test for the AI models constructed based on MRI for diagnosing ONFH.

**Supplementary Figure 8.** Fagan's nomogram for the AI models constructed based on MRI for diagnosing ONFH.

**Supplementary Figure 9.** Forest plot of sensitivity and specificity for the artificial intelligence models constructed based on ARCO staging for diagnosing ONFH.

**Supplementary Figure 10.** SROC curve for the AI models constructed based on ARCO staging for diagnosing ONFH.

**Supplementary Figure 11.** Deeks' test for the AI models constructed based on ARCO staging for diagnosing ONFH.

**Supplementary Figure 12.** Trim-and-fill plot for the AI models constructed based on ARCO staging for diagnosing ONFH.

**Supplementary Figure 13.** Fagan's nomogram for the AI models constructed based on ARCO staging for diagnosing ONFH.

**Supplementary Figure 14.** Forest plot of sensitivity and specificity for the AI models constructed based on other reference standards for diagnosing ONFH.

**Supplementary Figure 15.** SROC curve for the AI models constructed based on other reference standards for diagnosing ONFH.

**Supplementary Figure 16.** Deeks' test for the AI models constructed based on other reference standards for diagnosing ONFH.

**Supplementary Figure 17.** Fagan's nomogram for the AI models constructed based on other reference standards for diagnosing ONFH.

**Supplementary Figure 18.** Forest plot of sensitivity and specificity for the AI models constructed based on early-stage ONFH for diagnosing ONFH.

**Supplementary Figure 19.** SROC curve for the AI models constructed based on early-stage ONFH for diagnosing ONFH.

**Supplementary Figure 20.** Deeks' test for the AI models constructed based on early-stage ONFH for diagnosing ONFH.

**Supplementary Figure 21.** Fagan's nomogram for the AI models constructed based on early-stage ONFH for diagnosing ONFH.

**Supplementary Figure 22.** Forest plot of sensitivity and specificity for the AI models constructed based on all-stage ONFH for diagnosing ONFH.

**Supplementary Figure 23.** SROC curve for the AI models constructed based on all-stage ONFH for diagnosing ONFH.

**Supplementary Figure 24.** Deeks' test for the AI models constructed based on all-stage ONFH for diagnosing ONFH.

**Supplementary Figure 25.** Fagan's nomogram for the AI models constructed based on all-stage ONFH for diagnosing ONFH.

**Supplementary Figure 26.** Forest plot of sensitivity and specificity for the AI models constructed based on healthy controls for diagnosing ONFH.

**Supplementary Figure 27.** SROC curve for the AI models constructed based on healthy controls for diagnosing ONFH.

**Supplementary Figure 28.** Deeks' test for the AI models constructed based on healthy controls for diagnosing ONFH.

**Supplementary Figure 29.** Fagan's nomogram for the AI models constructed based on healthy controls for diagnosing ONFH.

**Supplementary Figure 30.** Forest plot of sensitivity and specificity for the AI models constructed based on disease controls for diagnosing ONFH.

**Supplementary Figure 31.** SROC curve for the AI models constructed based on disease controls for diagnosing ONFH.

**Supplementary Figure 32.** Deeks' test for the AI models constructed based on disease controls for diagnosing ONFH.

**Supplementary Figure 33.** Fagan's nomogram for the AI models constructed based on disease controls for diagnosing ONFH.

**Supplementary Figure 34.** Forest plot of sensitivity and specificity for the AI models constructed based on internal validation for diagnosing ONFH.

**Supplementary Figure 35.** SROC curve for the AI models constructed based on internal validation for diagnosing ONFH.

**Supplementary Figure 36.** Deeks' test for the AI models constructed based on internal validation for diagnosing ONFH.

**Supplementary Figure 37.** Trim-and-fill plot for the AI models constructed based on internal validation for diagnosing ONFH.

**Supplementary Figure 38.** Fagan's nomogram for the AI models constructed based on internal validation for diagnosing ONFH.

**Supplementary Figure 39.** Forest plot of sensitivity and specificity for the AI models constructed based on external validation for diagnosing ONFH.

**Supplementary Figure 40.** SROC curve for the AI models constructed based on external validation for diagnosing ONFH.

**Supplementary Figure 41.** Deeks' test for the AI models constructed based on external validation for diagnosing ONFH.

**Supplementary Figure 42.** Fagan's nomogram for the AI models constructed based on external validation for diagnosing ONFH.

**Supplementary Figure 43.** Forest plot of sensitivity and specificity for the AI models constructed based on single-center for diagnosing ONFH.

**Supplementary Figure 44.** SROC curve for the AI models constructed based on single-center for diagnosing ONFH.

**Supplementary Figure 45.** Deeks' test for the AI models constructed based on single-center for diagnosing ONFH.

**Supplementary Figure 46.** Trim-and-fill plot for the AI models constructed based on single-center for diagnosing ONFH.

**Supplementary Figure 47.** Fagan's nomogram for the AI models constructed based on single-center for diagnosing ONFH.

**Supplementary Figure 48.** Forest plot of sensitivity and specificity for the AI models constructed based on multi-center for diagnosing ONFH.

**Supplementary Figure 49.** SROC curve for the AI models constructed based on multi-center for diagnosing ONFH.

**Supplementary Figure 50.** Deeks' test for the AI models constructed based on multi-center for diagnosing ONFH.

**Supplementary Figure 51.** Trim-and-fill plot for the AI models constructed based on multi-center for diagnosing ONFH.

**Supplementary Figure 52.** Fagan's nomogram for the AI models constructed based on multi-center for diagnosing ONFH.

**Supplementary Figure 53.** Forest plot of sensitivity and specificity for the AI models constructed based on DL for diagnosing ONFH.

**Supplementary Figure 54.** SROC curve for the AI models constructed based on DL for diagnosing ONFH.

**Supplementary Figure 55.** Deeks' test for the AI models constructed based on DL for diagnosing ONFH.

**Supplementary Figure 56.** Trim-and-fill plot for the AI models constructed based on DL for diagnosing ONFH.

**Supplementary Figure 57.** Fagan's nomogram for the AI models constructed based on DL for diagnosing ONFH.
